# Neonatal *Elizabethkingia anophelis* meningitis originating from the water reservoir of an automated infant milk dispenser, the Netherlands, February 2024

**DOI:** 10.2807/1560-7917.ES.2024.29.14.2400177

**Published:** 2024-04-04

**Authors:** B Ruben Brandsema, Ger-Jan Fleurke, Sigrid Rosema, Eke MW Schins, Jelte Helfferich, Erik Bathoorn

**Affiliations:** 1 University Medical Center Groningen, Department of Paediatric Infectious Diseases and Immunity, Groningen, the Netherlands; 2 University Medical Center Groningen, Department of Medical Microbiology, Groningen, the Netherlands; 3 University Medical Center Groningen, Department of Neonatology, Groningen, the Netherlands; 4 University Medical Center Groningen, Department of Neurology, Groningen, the Netherlands; * These authors contributed equally to this work and share first/last authorship.

**Keywords:** *Elizabethkingia anophelis*, meningitis, molecular epidemiology, surveillance

## Abstract

*Elizabethkingia anophelis* is a multidrug-resistant pathogen causing high mortality and morbidity in adults with comorbidities and neonates. We report a Dutch case of *E. anophelis* meningitis in a neonate, clonally related to samples taken from an automated infant milk dispenser located at the family’s residence. We inform about the emergence of *E. anophelis* and suggest molecular surveillance in hospitals and other health settings. This is the first case connecting an automated formula dispenser to an invasive infection in a neonate.

Here we report a neonate in the Netherlands hospitalised for treatment of a community-acquired meningitis caused by *Elizabethkingia anophelis*. Analysis by core genome multilocus sequence typing (cgMLST) based on the core genome sequence of the *E. anophelis* strain isolated from cerebrospinal fluid (CSF) showed that it was clonally related to samples of the water reservoir of an automated infant milk dispenser that the family had used at home. This household appliance contains reservoirs for both water and infant formula, to automatically prepare a warmed milk bottle for infants.

We wish to raise awareness about a possible emergence of this pathogen in the community. This bacterium has already caused outbreaks of severe, difficult-to-treat infections in other parts of the world [1,2].

## Case description

A previously well 8-day-old newborn was admitted to a secondary hospital with feeding difficulties, grunting and a fever of 38.7 °C. Physical examination showed normal respiratory and circulatory parameters, but notable irritability. With suspected sepsis and meningitis, additional tests were performed. Blood tests showed no leukocytosis but elevated C-reactive protein at 222 mg/L (normal range: < 5 mg/L). The CSF showed marked pleocytosis with leukocytes at 2,368 × 10^6^/L (87% polynuclear) (normal range: < 16 × 10^6^/L), glucose at 2.6 mmol/L (normal range: 1.9–5.6 mmol/L) and protein at 1,502 mg/L (normal range: 510–1,010 mg/L). Cultures of both blood and CSF were done, and empirical treatment for meningitis was started with amoxicillin and cefotaxime. On the next day, both cultures showed growth of *E. anophelis* (determined by MALDI-TOF, Bruker, Germany). The antibiotic regimen was switched to trimethoprim/sulfametrole (TMP/SMZ) 9 mg/kg/day and ciprofloxacin 30 mg/kg/day intravenously pending susceptibility results. Cefotaxime was discontinued because of intrinsic resistance of *E. anophelis* against cephalosporins [3].

Antimicrobial susceptibility testing was performed using E-Test (bioMérieux, Marcy-l'Étoile, France), testing for trimethoprim/sulfamethoxazole, quinolones, tetracyclines, aminoglycosides, carbapenems, rifampicin and vancomycin. The strain was also sent to the Dutch reference laboratory (Radboudumc, Department of Medical Microbiology, Nijmegen, The Netherlands) for broth microdilution. The patient was transferred to our neonatal intensive care unit for monitoring and possible neurosurgical intervention. Magnetic resonance imaging of the brain showed leptomeningeal enhancement, ventriculitis, hydrocephalus and adhesions caudal of the fourth ventricle, but no abscess or empyema. On day 7 of admission, ciprofloxacin was replaced with moxifloxacin 5 mg/kg/day once daily after the E-test indicated ciprofloxacin resistance, later confirmed with broth microdilution. 

The patient initially recovered, with complete normalisation of inflammatory markers. However, progressive, non-communicating hydrocephalus required the placement of an Ommaya reservoir on day 14. Cultures taken from the CSF on day 19 still grew *E. anophelis*. Antimicrobial susceptibility testing on this sample was performed with the methods mentioned above. Based on the initial susceptibility test results in the E-Test, TMP/SMZ treatment was continued. Moxifloxacin was increased to 10–12 mg/kg/day under pharmacokinetic/pharmacodynamic (PK/PD) monitoring, due to an increase in minimum inhibitory concentration (MIC) increase from 0.25 to 1 mg/L. We added rifampicin 20 mg/kg/day i.v. and vancomycin 5 mg/day i.t. (under PK/PD monitoring). The CSF samples were sterile after day 22. The child is currently clinically stable awaiting definite treatment for the hydrocephalus.

## Environmental investigation

The treating clinicians performed extensive interviews with the parents on possible contaminated water supplies; these revealed that they had fed the child with milk bottles prepared by an automated formula dispenser. They provided the machine to our hospital on day 15, still containing water in the reservoir from the day of initial hospital admission. We took cultures from water and biofilm (eSwab, MLS, Menen, Belgium) of the bottom of the reservoir. Both grew *E. anophelis*, later confirmed to be clonally related to the isolate from the patient. Therefore, on day 22, we contacted the Netherlands Food and Consumer Product Safety Authority (NVWA), the National Institute for Public Health and the Environment, and the vendor of the machine. The vendor stopped the sales of this specific machine, and NVWA issued a recall by contacting consumers personally via the vendor. The NVWA also provided a reservoir of an unused machine to our hospital. We sampled that water reservoir and formula compartment (eSwab, MLS, Menen, Belgium), which tested negative. We also cultured water the parents provided from their faucet, which had been used to fill the water reservoir of their machine. This sample was provided on day 20 of admission and showed no growth. 

## Antimicrobial susceptibility 

The Table presents the susceptibility profiles of the initial clinical isolate (2408_M1) and the isolate from day 19 (2408_M2). We interpreted susceptibility according to the European Committee on Antimicrobial Susceptibility Testing (EUCAST) guidance document *When there are no breakpoints in breakpoint tables* [4]. The tetracycline susceptibility, not mentioned in the EUCAST guidance document, was interpreted according to Clinical and Laboratory Standards Institute (CLSI) breakpoints [5]. Susceptibility results of the isolates suggested that treating the patient with intravenous carbapenems, aminoglycosides, vancomycin and colistin should be discouraged [4]. The clinical isolates showed low MICs for TMP/SMZ, minocycline, moxifloxacin and rifampicin. To explore potential factors contributing to the microbiological relapse we additionally tested the isolates for trimethoprim and levofloxacin susceptibility; both isolates showed resistance to trimethoprim, and isolate 2408_M2 had an increased levofloxacin MIC. 

**Table:** Minimum inhibitory concentrations and interpretations of two *Elizabethkingia anophelis* isolates, the Netherlands, February 2024

Antimicrobial drug	Day 0		Day 19	
	MIC in mg/L^a^	Interpretation^b^	MIC in mg/L^a^	Interpretation^b^
Trimethoprim/sulfamethoxazole	0.5	CFT	> 4	R
Ciprofloxacin	2	DFT	> 8	R
Moxifloxacin	0.25	CFT	2	DFT
Levofloxacin	0.5^c^	CFT	3^c^	DFT
Doxycycline	1	S^d^	8	I^d^
Minocycline	0.25	S^d^	1	S^d^
Tigecycline	1	NA	1	NA
Clarithromycin	4	NA	8	NA
Tobramycin	> 16	R	> 16	R
Amikacin	64	R	16	R
Imipenem	32	R	> 32	R
Rifampicin	0.5	NA	1	NA
Colistin	> 16	R	ND	
Vancomycin	12^c^	NA	8^c^	NA

CFT: considered for treatment (formal categorising of the susceptibility of the organism is not possible; cautious interpretation suggests that the agent may be considered for therapy); DFT: discouraged for treatment (formal categorising of the susceptibility of the organism is not possible; the MIC suggests that the agent should not be used for therapy); I: intermediate; MIC: minimum inhibitory concentration; NA: not applicable (due to lack of breakpoints for interpretation); ND: not determined; R: resistant; S: susceptible.

^a^ Measured by broth microdilution unless otherwise specified.

^b^ Interpretation according to [4].

^c^ Measured by E-test (bioMérieux).

^d^ Interpretation according to CLSI breakpoints for Other non-Enterobacterales (M100) [5].

## Molecular comparison

The clinical and water reservoir isolates were compared by cgMLST, based on short-read sequencing as described previously [6]. The isolates were identical using an ad hoc scheme, based on reference genomes, including 2,699 target genes. The Figure presents a core-genome single nucleotide variant (SNV)-based neighbour-joining tree inferred from 194,435 nt, confirming the species determination of *E. anophelis*. The isolates have no close matches with any of the sequences publicly available in the National Center for Biotechnology Information (NCBI) database. A previous Dutch clinical *E. anophelis* isolate was identified within the same lineage, but not suspected of recent transmission. The isolates belonging to this lineage are globally distributed, including isolates from France, Sweden, the United Kingdom, Canada, the United States and countries in Asia. We detected no mutations in the genes *gyrA*, *grlA*, *grlB* associated with fluoroquinolone resistance.

**Figure:**
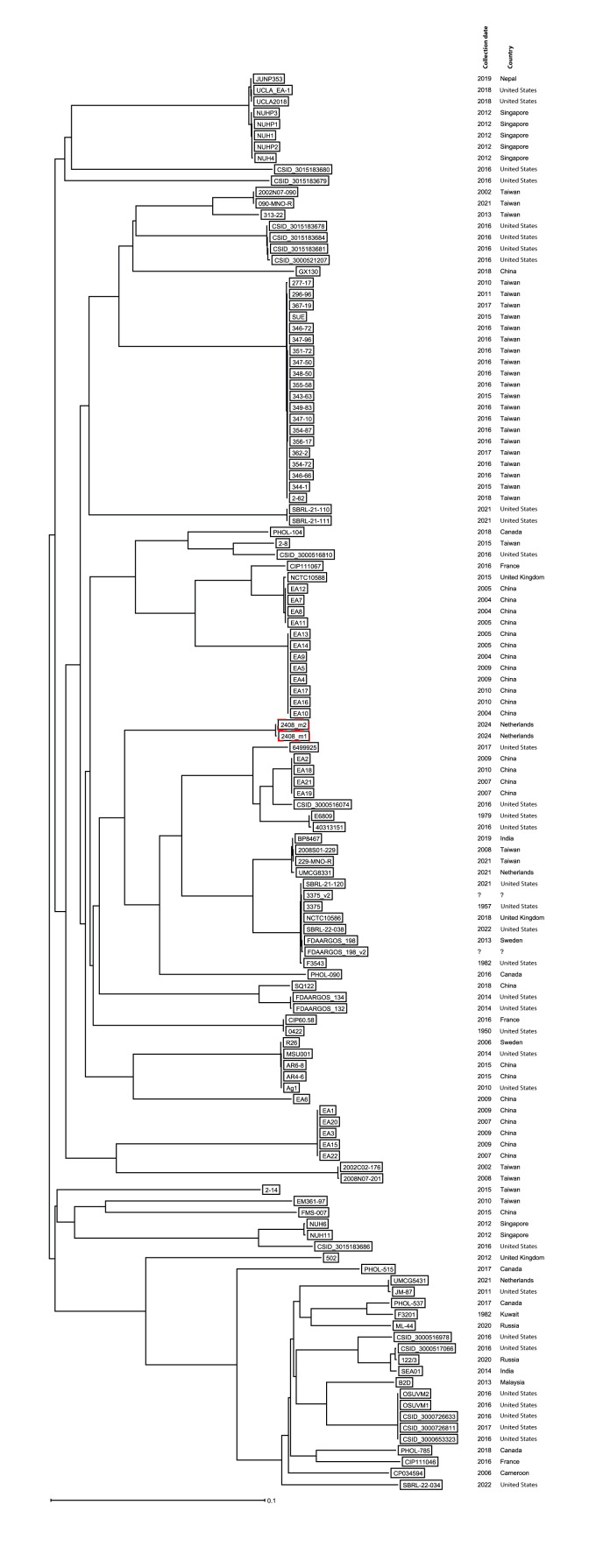
Neighbour-joining tree of *Elizabethkingia anophelis* isolates (n = 125)

The tree is based on 194,435 nt, pairwise ignoring missing values.

## Discussion

*Elizabethkingia anophelis* are Gram-negative rod-shaped bacteria commonly detected in soil and wastewater. Invasive infections often cause severe disease, particularly in neonates and immunocompromised patients. The pathogen causes meningitis, bacteraemia and pneumonia [7,8]. Treatment of infections is challenging since *E. anophelis* is intrinsically resistant to multiple antibiotic drug classes including carbapenems [3]. Because of its limited antimicrobial susceptibility and high pathogenicity in neonates, older adults and immunocompromised people, treatment outcomes are often poor. Neonatal meningitis in particular is associated with high mortality (33%) or persistent neurological damage, mostly because of progressive hydrocephalus (50%) [9]. In Asia and the United States, extensive outbreaks with *E. anophelis* have been reported in hospitals and the community in the past decade [10,11]. Contaminated water taps are the most common cause of *E. anophelis* outbreaks. However, a source can remain unidentified for a long time given the low infection rates in healthy individuals [12].

We have presented a case of community-acquired severe meningitis due to *E. anophelis* in the Netherlands. So far, *E. anophelis* have scarcely been reported in Europe. In 2020 and 2021, a community-acquired clonal outbreak occurred in France including 20 cases, nine of which were fatal. Despite environmental source investigation, the origin of the outbreak could not be identified [1]. In addition, nosocomial transmission through a lung transplant was described in the Netherlands in 2022. The identity of the donor was confidential, so no source investigation of the index patient was possible [12]. The largest outbreak so far comprised 63 confirmed cases in the United States of whom 18 were fatal. Even in that large, predominantly community-acquired, clonal outbreak, a source could not be identified [2]. The authors of the study therefore urgently recommended laboratory-based surveillance of *E. anophelis* isolates from healthcare institutions.

Treatment failure and high fatality rates are often reported in *E. anophelis* infections. The antibiotic treatment options in neonatal meningitis are limited due to this pathogen’s extensive intrinsic resistance, limited options with good CSF penetrance, and toxicity issues in neonates. In addition, *E. anophelis* is highly adaptive, and quinolone treatment tends to select for mutations [13]. In our case, the combined treatment of moxifloxacin with TMP/SMZ did not prevent the selection of levofloxacin resistance in the second CSF isolate. The resistance to the trimethoprim component could have been a contributing factor in the patient’s relapse since this hampers the bactericidal activity of TMP/SMZ.

We detected *E. anophelis* in a water sample and a biofilm sample taken from the reservoir of an automated formula dispenser. Cultures from residential tap water and an unused machine showed no signs of *E. anophelis.* We are therefore unsure how the reservoir had been contaminated. Recalled machines from the homes of consumers will be investigated in our hospital in the future. This is the first reported case of an invasive neonatal infection connected to the use of an automated formula dispenser.

The main limitation of this study is that the source of contamination of the automated formula dispenser has not been fully elucidated. The water network of the patient’s residence was sampled only once from a single tap point but has not yet been thoroughly surveyed. There was no indication of *E. anophelis *emergence in routinely performed water surveillance in The Netherlands. 

In addition, there has not been an increase in patient samples with *E. anophelis* in the Netherlands between 2018 and 2023 (personal communication, National Institute for Public Health and the Environment). We have chosen to report this case nonetheless, to increase the awareness and improve management in case of an outbreak. 

## Conclusion

We wish to raise awareness about the emergence of *E. anophelis* and propose that molecular surveillance should be considered in hospitals and other health settings. Given the fact that we could not identify the original source of contamination, we cannot rule out that more cases might occur in the future. Rapid identification of the spread of *E. anophelis* and elimination of the source could prevent large-scale outbreaks.
